# Supplementary figures and images for: SIRT-1 is required for release of enveloped enteroviruses
Source: eLife. 2023 Oct 18;12:RP87993. doi: 10.7554/eLife.87993 (PMC10584371; doi:10.7554/eLife.87993)

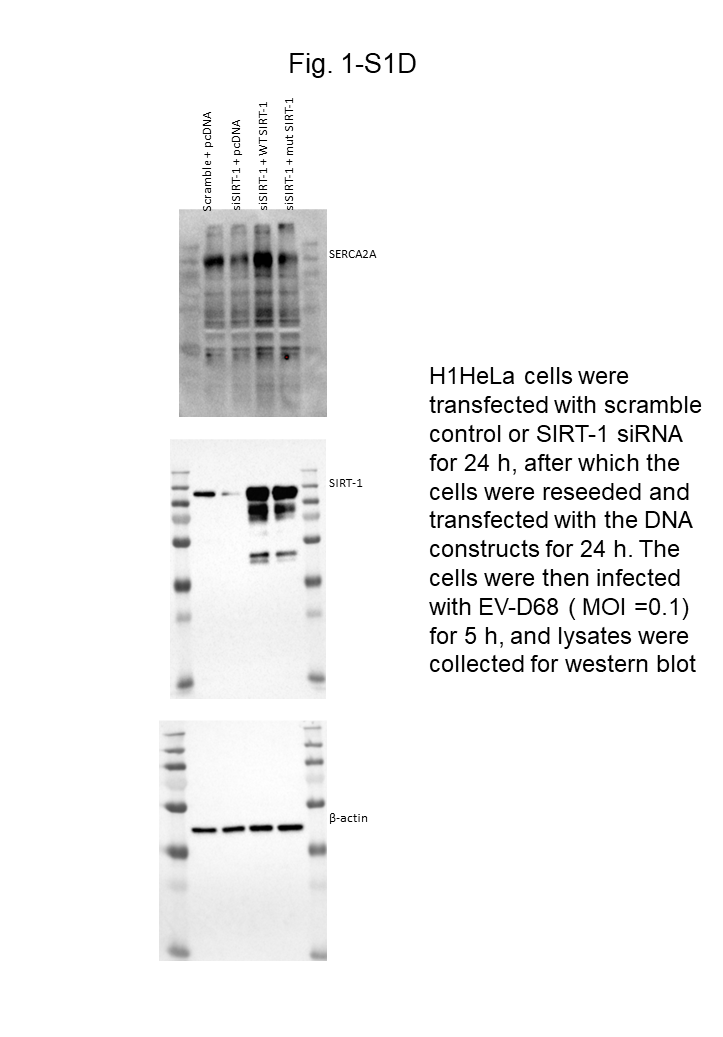

Supplement: Source data 1. [file elife-87993-data1.zip › Figure 1-figure supplement 1-source data 1.tif]

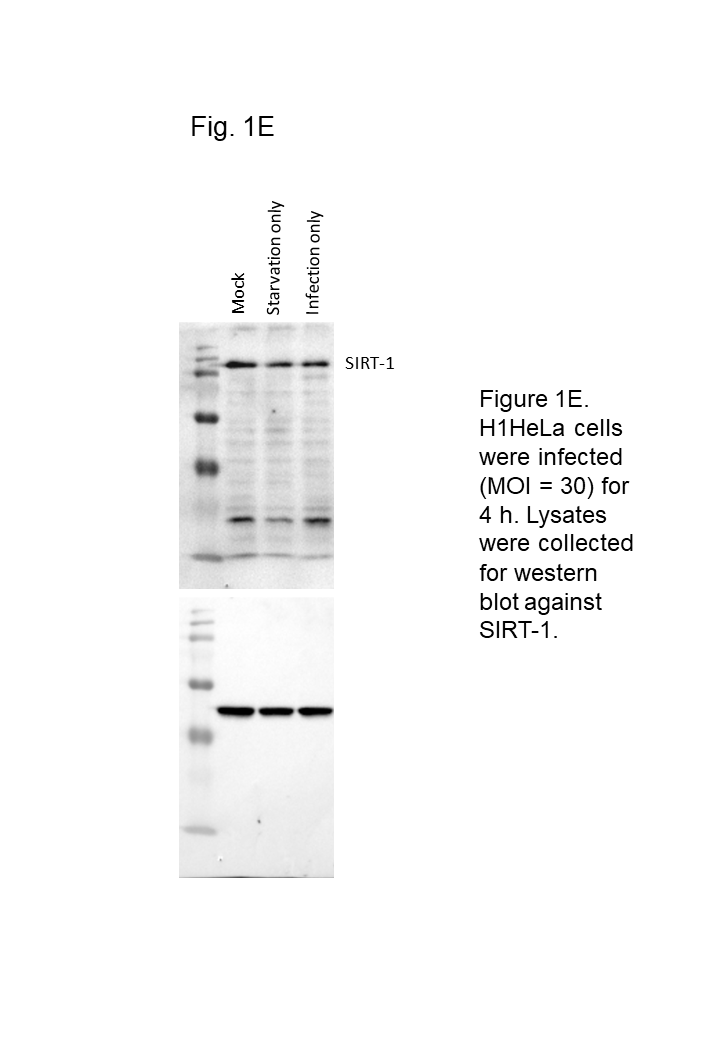

Supplement: Source data 1. [file elife-87993-data1.zip › Figure1 - source data 1.TIF]

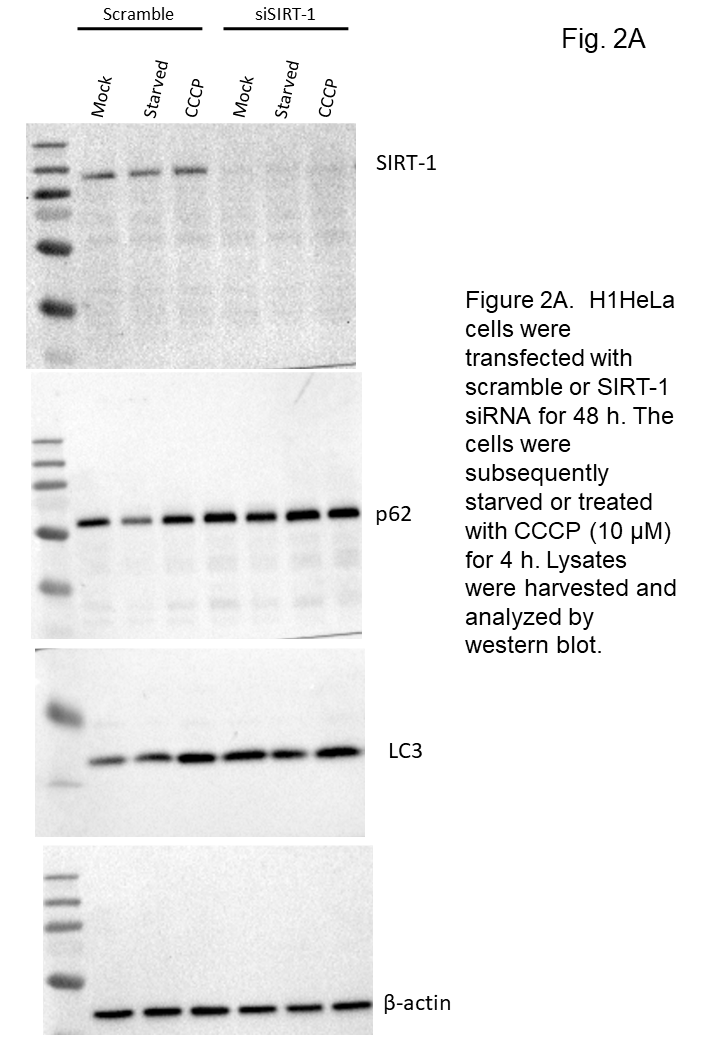

Supplement: Source data 1. [file elife-87993-data1.zip › Figure2 - source data 1.TIF]

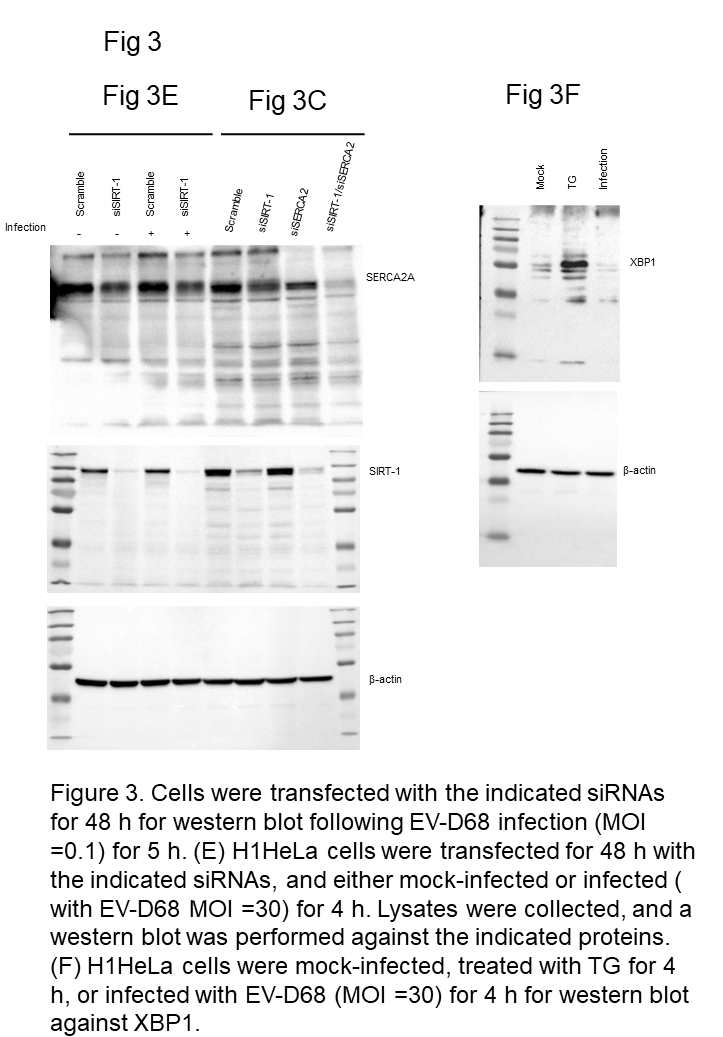

Supplement: Source data 1. [file elife-87993-data1.zip › Figure3 - source data 1.TIF]

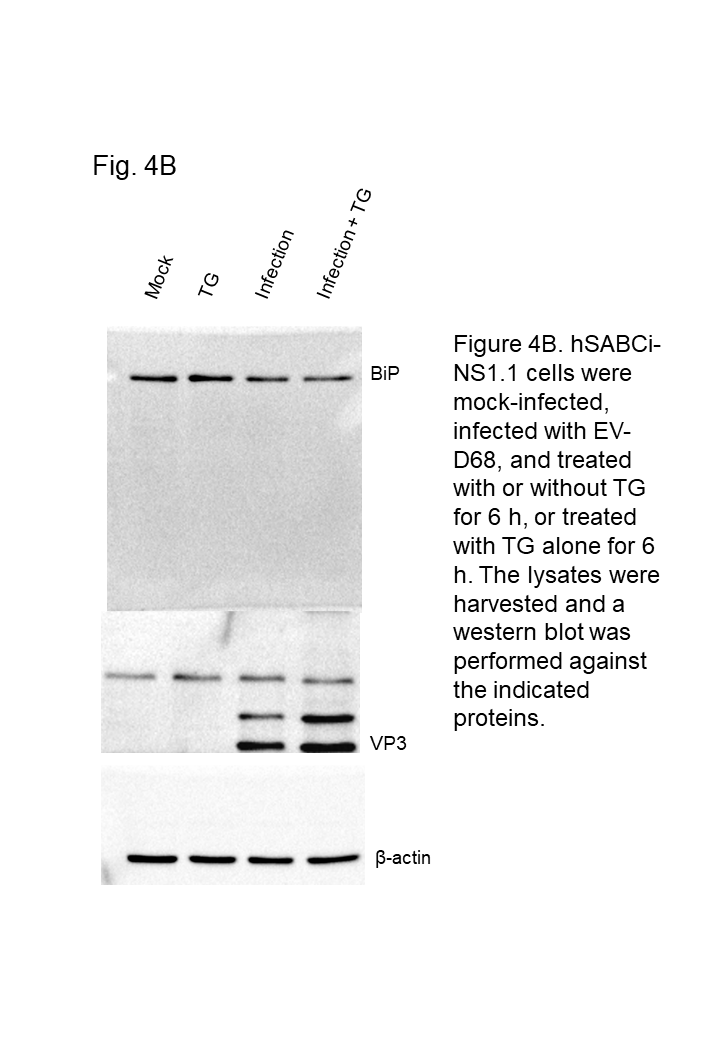

Supplement: Source data 1. [file elife-87993-data1.zip › Figure4 - source data 1.TIF]

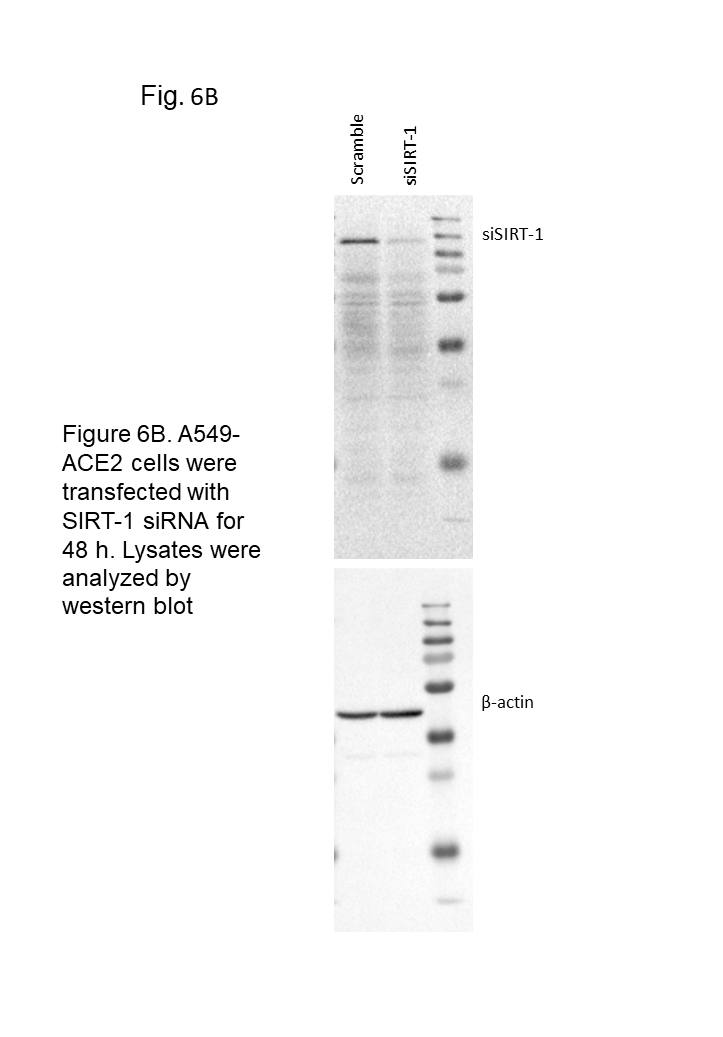

Supplement: Source data 1. [file elife-87993-data1.zip › Figure6 - source data 1.TIF]

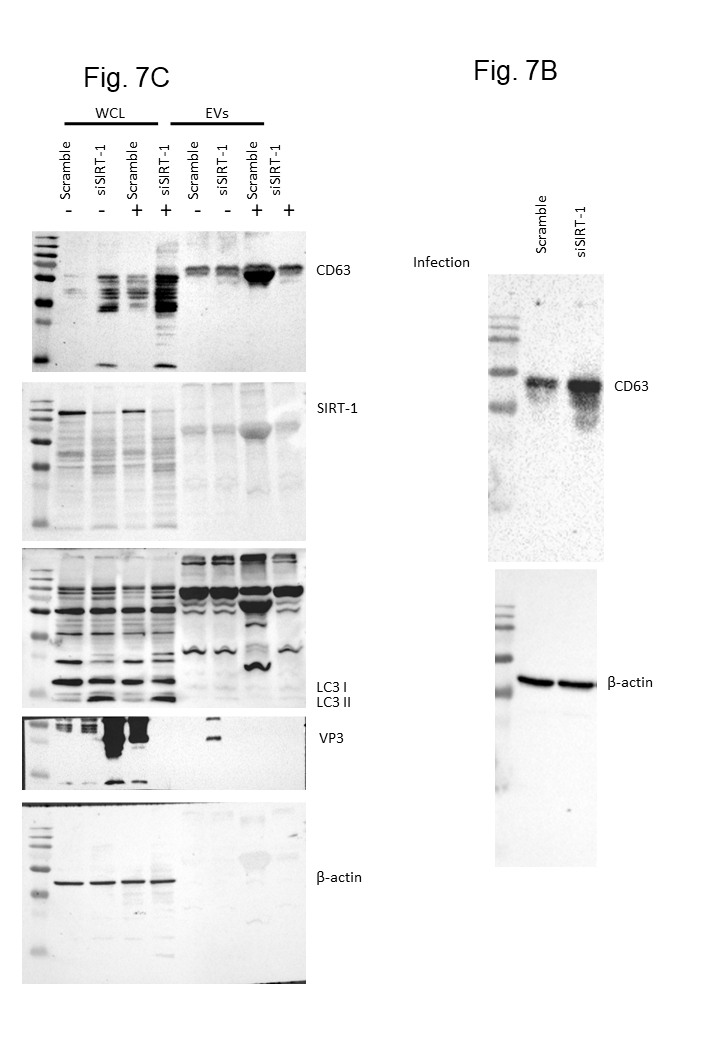

Supplement: Source data 1. [file elife-87993-data1.zip › Figure7 - source data 1.TIF]
